# Enhanced Production of Docosahexaenoic Acid in Mammalian Cells

**DOI:** 10.1371/journal.pone.0096503

**Published:** 2014-05-02

**Authors:** Guiming Zhu, Xudong Jiang, Qin Ou, Tao Zhang, Mingfu Wang, Guozhi Sun, Zhao Wang, Jie Sun, Tangdong Ge

**Affiliations:** Laboratory of Biochemistry and Molecular Biology, College of Basic Medicine, Jiamusi University, Jiamusi, Heilongjiang, China; National Institute of Nutrition, India

## Abstract

Docosahexaenoic acid (DHA), one of the important polyunsaturated fatty acids (PUFA) with pharmaceutical and nutraceutical effects, may be obtained through diet or synthesized in vivo from dietary *a*-linolenic acid (ALA). However, the acumulation of DHA in human body or other mammals relies on the intake of high dose of DHA for a certain period of time, and the bioconversion of dietary ALA to DHA is very limited. Therefore the mammalian cells are not rich in DHA. Here, we report a new technology for increased prodution of DHA in mammalian cells. By using transient transfection method, *Siganus canaliculatus* Δ4 desaturase was heterologously expressed in chinese hamster ovary (CHO) cells, and simultaneously, mouse Δ6-desaturase and Δ5-desaturase were overexpressed. The results demonstrated that the overexpression of Δ6/Δ5-desaturases significantly enhanced the ability of transfected cells to convert the added ALA to docosapentaenoic acid (DPA) which in turn get converted into DHA directly and efficiently by the heterologously expressed Δ4 desaturase. This technology provides the basis for potential utility of these gene constructs in the creation of transgenic livestock for increased production of DHA/related products to meet the growing demand of this important PUFA.

## Introduction

Docosahexaenoic acid (DHA; 22∶6n-3) is an n-3 polyunsaturated fatty acid (PUFA) that is particularly enriched in mammalian tissues such as brain, testis, and retina. Clinical studies have demonstrated the crucial role of DHA in the development and functions of these tissues [1], [2]. DHA enhances membrane elasticity and molecular motion and thus promotes signal transduction via enhanced protein/receptor interactions [3]–[6]. DHA is also the activating ligand for multiple transcriptional factors that control the expression of enzymes involved in fatty acid synthesis and β-oxidation [7]. Depletion of brain DHA in some mammals leads to distinct impairments in brain and neural function [8]–[13]. In adult humans, low levels of DHA in blood have been correlated with psychological disturbances such as alcoholism, major depression (nonpsychotic), postpartum depression, and senile dementia [19]–[21]. The essentiality of DHA for human infant nutrition in support of neuronal function has been shown by DHA supplementation, enhancing visual acuity and cognition-related test scores in human infants [14]–[18].

It is well established that DHA can be biosynthesized from α-linolenic acid (ALA,18∶3*n*-3), a shorter chain *n*-3 fatty acid precursor, through chain elongation and desaturation processes. α-linolenic acid is desaturated to 18∶4*n*-3 by Δ6-desaturase, chain-elongated to 20∶4*n*-3, and subsequently converted to eicosapentaenoic acid (EPA, 20∶5*n*-3), also a biologically active n-3 fatty acids, by Δ5-desaturase in the endoplasmic reticulum. Mammalian Δ5- and Δ6-desaturases have been identified and cloned [22]. However, Δ4-desaturase, responsible for making 22∶6*n*-3 directly from docosapentaenoic acid (DPA, 22∶5n-3), an elongation product of 20∶5*n*-3, has been identified only in a few of organisms such as microalgae [23]. In mammals, 22∶5n-3 is further elongated to 24∶5*n*-3 followed by desaturation by Δ6-desaturase to 24∶6*n*-3. Subsequently, 24∶6n-3 is transferred to peroxisomes and converted to 22∶6n-3 by removing two carbon chains by β-oxidation. Long-chain n-6 fatty acids, such as arachidonic acid (ARA,20∶4n-6), the commonly observed *n*-6 fatty acids in most tissues, are biosynthesized from linoleic acid (LA, 18∶2n-6) using the analogous pathway and the same enzyme system.

Although dietary ALA can be converted to DHA in vivo, the efficiency of conversion is limited [24]. It was shown that the mature rat brain has little capacity to synthesize its own DHA, even when stimulated to do so, under dietary conditions of feeding ALA alone or during n-3 PUFA dietary deprivation [25], [26]. In contrast, the adult rat liver does convert ALA to DHA, but at a rate sufficient to convert <2% of the total ALA that enters the liver per unit time, with the majority (>70%) of the entering ALA being lost to β-oxidation. Therefore, mammals are unable to produce high levels of DHA in their bodies. Algae are the primary producers of DHA and EPA in the ecosystem, and several refined algal oils are rich sources of DHA. Fish consume micro algae (phyto plankton) and therefore are rich in DHA and EPA, thus fish become the main dietary source of these PUFA. Nevertheless, marine products such as encapsulated fish oil are not suitable for daily use. Furthermore, the decline in marine fish stocks and the potential contamination of marine products with mercury and other chemicals call for land-based dietary sources of DHA. One strategy is to create genetically modified land animals by making use of the desaturases and elongases involved in DHA biosynthesis. In this paper, we report the successful production of DHA in chinese hamster ovary (CHO) cells by heterologous expression of *Siganus canaliculatus* Δ4 desaturases after overexpression of mice Δ6/Δ5-desaturase. The results provide a basis for potential applications of this technology in creating of transgenic livestock for enhanced production of DHA in the related products.

## Materials and Methods

### Construction of Recombinant Plasmids

The Δ4 desaturase gene from *Siganus canaliculatus*, *sScD4*, was artificially synthesized (with genetic codons optimized) from its original sequence (GenBank accession number: GU594278). Then, the synthetic gene (1352 bp) was inserted into the pcDNA3.1 expression vector with EcoRI/HindIII double digestion. The expression vectors for *fads2* and *fads1*genes (cDNAs), coding for mouse Δ6/-desaturase and Δ5-desaturase respectively, were constructed previously in our laboratory, with the cDNAs cloned into pcDNA3.1. The double-gene expression vector pcDNA3.1-F2F1 was constructed as follows: based on the enzyme sites BglII/MluIcarried on pcDNA3.1-fad1, PCR primers were designed to amplify the pCMV-fad2-polyA DNA fragment on pcDNA3.1-fad2, and then the amplicon was inserted into pcDNA3.1- fad1 by BglII/MluIdouble digestion and ligation.

### Cell Cultures and transfection

Transient transfections in chinese hamster ovary (CHO) cells were carried out using Lipofectamine™ 2000 (Invitrogen Inc., USA) as described in the manufacturer's manual. One day prior to transfection, cells were plated onto 60-mm culture dishes in Dulbecco's modified Eagle's medium (DMEM) supplemented with 10% fetal bovine serum (FBS) and 10 µM PUFA substrates (DPA for sScD4 transfections, LA and ALA for F2F1 transfections). Cells were transfected with 8 µg of the plasmids. To monitor transfection efficiencies, cells were transfected with an expression vector containing the enhanced green fluorescent protein (EGFP) cDNA, pcDNA3.1-EGFP. The day following transfection, media were replaced with fresh media containing the same PUFA substrates as above. Two days after transfection, cells were harvested and used for analysis of gene expression or fatty acid composition.

### RT-PCR

RT-PCR was performed to analyze transcripts in transgenic cells.Total RNA was extracted from the harvested cells, using TRIzol Reagent (Invitrogen) followed by DNase treatment to remove genomic DNA contamination. cDNA was prepared using the TaKaRa RNA PCR Kit 3.0(TaKaRa). The following primer pairs were used to detect the target genes—sScD4: 5′- TCGGGCATCTGAGCGTGTTC- 3′ and 5′- ATACTCCACGGGCTGGGTCTCC- 3′; F2: 5′- TAGTCTAGAGCATGGGGAAGGGAGGTAAC - 3′and 5′-AGCAAGCTTCATTTATGGAGGT AAGCATCCAG - 3′; F1: 5′ TACTCTAGAGCTTGCTATGGCTCCCGACC -3′, 5′- ATGAAGCTTGCTATTGGTGAAGGTAAGCGTCC-3′. The PCR conditions were as follows: 94°C for 2 min for desaturation, 35 cycles of 94°C for 30 s for desaturation, 60°C for 30 s for annealing, and 72°C for 30 s for extension, followed by a final extension period of 72°C for 10 min. The amplified products were subjected to electrophoresis on a 2% agarose gel. The amplification of beta-actin gene was carried out as control.

### Lipid Analysis

The fatty acid composition of total cellular lipids was analyzed as following: an aliquot of cell pellet homogenate in a micro reaction vessel (Supelco) was mixed with 1 ml of 14% BF3/MeOH reagent containing 0.005% butylated hydroxytoluene (as antioxidant). After blanketed with nitrogen, the mixture was heated at 95–100°C for 1 h, then cooled to room temperature and methyl esters extracted in the hexane phase following addition of 1 ml H_2_O and 1 ml of hexane. The samples were centrifuged for 5 min at 3000 rpm, then the upper hexane layer was removed and concentrated under nitrogen. Fatty acid methyl esters were quantified with GC-MS by using an HP-5890 Series II gas chromatograph equipped with an Omegawax 250 capillary column (Supelco, Bellefonte, PA) attached to an HP-5971 mass spectrometer. The oven program was maintained initially at 150°C for 2 min, then ramped to 200°C at 10°C/min and held for 4 min, ramped again at 5°C/min to 240°C, held for 3 min, and finally, ramped to 270°C at 10°C/min and maintained for 5 min. Peaks were identified by comparison with fatty acid standards (Sigma), then area and percentage for each resolved peak were analyzed.

### Statistical Analysis

Statistical analysis was performed with SPSS version 18.0 for Windows, using one-way ANOVA followed by LSD comparison test for posthoc analyses. Results were expressed as the mean ± SD. A p-value of <0.05 was considered to be statistically significant.

## Results

**Figure 1:**
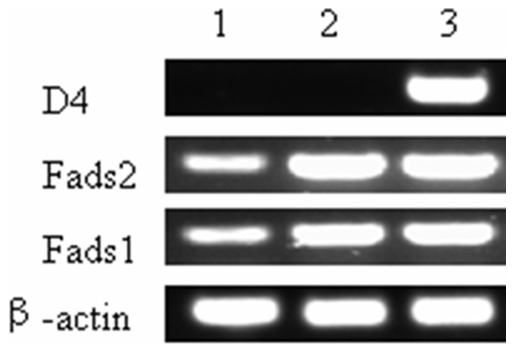
*sScD4*, *fads2* and *fads1* transcripts in transfected cells were analyzed by RT-PCR. Lane 1 were cells transfected with pcDNA3.1-*EGFP*; Lane 2 were cells transfected with pcDNA3.1-F2F1; Lane 3 were cells co-transfected with pcDNA3.1- F2F1 and pcDNA3.1-sScD4.

**Figure 2:**
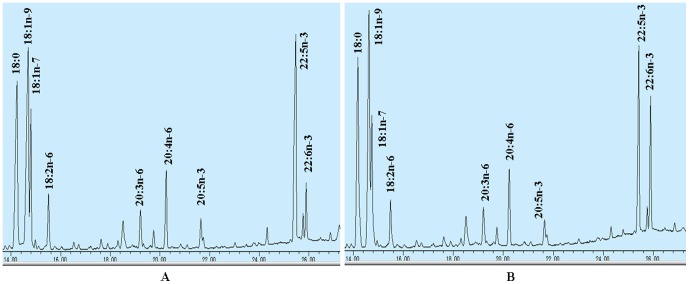
Partial gas chromatograph traces showing fatty acid profiles of total cellular lipids extracted from the control cells transfected with pcDNA3.1-EGFP (A), and the cells infected with pcDNA3.1-sScD4 (B).

To test whether pcDNA3.1-sScD4 could be expressed in mammalian cells, the vector was introduced into CHO cells, by using liposome transfection method. DPA was added to the cell culture medium for 3 days. The RT-PCR results showed mRNA of this synthetic gene could be greatly transcribed, while the cells transfected with the control plasmid pcDNA3.1-EGFP, which carries the enhanced green fluorescent protein as reporter gene, detected no *sScD4* mRNA (Fig.1). The transient transfections of pcDNA3.1-sScD4 in CHO cells confirmed *sScD4* can directly and efficiently convert DPA to DHA, as shown in Fig 2. Table 1 shows fatty acids composition of total cellular lipids from the CHO cells transfected with *EGFP* or *sScD4*. None of the percentage distribution of a fatty acid varied between the CHO cells transfected with *EGFP* and *sScD4* except DPA and DHA. The percentage distribution of DPA decreased from 20.2±1.62 in CHO cells transfected with *EGFP* to 14.4±1.46 in the cells transfected with *sScD4*, while the percentage distribution of DHA increased from 4.0±0.95 in cells transfected with *EGFP* to 9.2±0.94 in the cells transfected with *sScD4*.

**Table 1:** PUFA composition of total cellular lipids from the CHO cells transfected with EGFP or sScD4 genes.

Mol % of fatty acids	EGFP	sScD4
14∶0	4.6±0.42	4.5±0.31
16∶0	2.1±0.34	1.6±0.22
18∶0	19.2±2.13	20.3±2.51
18∶1n-9	24.4±3.35	23.6±2.87
18∶1n-7	9.1±1.86	10.9±1.94
18∶2n-6	4.2±0.93	4.5±1.17
20∶3n-6	1.4±0.38	1.6±0.35
20∶4n-6	5.8±1.69	6.3±2.12
20∶5n-3	3.3±0.89	2.3±0.81
22∶5n-3	20.2±1.62^a^	14.4±1.46^b^
22∶6n-3	4.0±0.95^b^	9.2±0.94^a^

Values are means of three measurements. Values for each fatty acid with different superscripts differ significantly (*P*<0.05) between control (EGFP), sEgD4 and sScD4.

**Figure 3:**
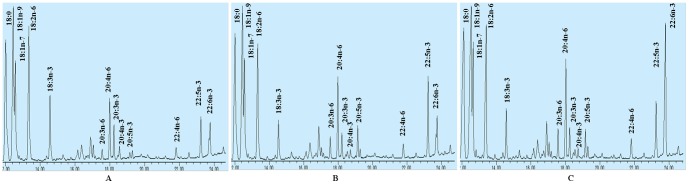
Partial gas chromatograph traces showing fatty acid profiles of total cellular lipids extracted from the control cells transfected with pcDNA3.1-EGFP (A), the cells transfected with pcDNA3.1- F2F1 (B) and the cells co-transfected with pcDNA3.1- F2F1 and pcDNA3.1-sScD4 (C).

CHO cells transfected with pcDNA3.1-F2F1 have the ability to overexpress of *fads2* and *fads1* genes as shown in Fig.1. The fatty acid profiles were remarkably different between the control cells transfected with pcDNA3.1-EGFP and the cells transfected with pcDNA3.1- F2F1(Fig.3). In the cells overexpressing the Δ6/Δ5 desaturase, the percentage distribution of 18∶2*n*-6 and 18∶3*n*-3 were significantly decreased, while 20∶3*n*-6, 20∶4*n*-6, 20∶5*n*-3, 22∶5*n*-3 were significantly increased, as compared to the control cells. This means the substrates (18∶2*n*-6 and 18∶3*n*-3) added to the transfected cells were efficiently converted into longer PUFA. However, the DHA level increased slightly but was not statistically significant, indicating that the overexpression of the Δ6/Δ5 desaturases did not contribute to the elevation of DHA level. An interesting phenomenon is that the percentage composition of 20∶3*n*-3 and 20∶4*n*-3 were also significantly decreased in the cells overexpressing the Δ6/Δ5 desaturase when compared to the control cells. Obviously, the cells tend to convert them to longer chain PUFA, such as 20∶5*n*-3, 22∶5*n*-3.

The co-transfection of pcDNA3.1-F2F1 and pcDNA3.1-sScD4 into CHO cells also showed similar overexpression of *fads2* and *fads1* genes as cells transfected with pcDNA3.1-F2F1, but only the former detected the expression of *sScD4* gene (Fig.1). The Δ4 desaturase (*sScD4* gene product) confer the cells co-transfected with pcDNA3.1-F2F1 and pcDNA3.1-sScD4 the ability of efficiently converting the accumulated DPA to DHA (Fig.3). The level of DHA in the cells co-transfected with pcDNA3.1-F2F1 and pcDNA3.1-sScD4 was more than twofold higher than in the cells transfected with pcDNA3.1-F2F1, and threefold higher than in the cells transfected with pcDNA3.1-EGFP (Table 2). In addition, compared to the cells transfected with pcDNA3.1-F2F1, the percentage distribution of 20∶5*n*-3, 22∶5*n*-3 in CHO cells co-transfection of pcDNA3.1-F2F1 and pcDNA3.1-sScD4 decreased significantly in that they were converted to DHA.

**Table 2:** PUFA composition of total cellular lipids from the CHO cells transfected with *EGFP*, *F2F1* or *F2F1+D4*.

Mol % of fatty acids	EGFP	F2F1	F2F1+D4
14∶0	4.2±0.83	3.8±0.81	3.2±0.78
16∶0	0.7±0.03	0.8±0.04	2.1±0.11
18∶0	18.8±2.12	19.6±2.33	18.2±1.98
18∶1n-9	20.3±1.18	20.3±1.12	20.7±1.27
18∶1n-7	10.9±0.91	10.3±0.95	8.2±0.78
n-6 PUFA			
18∶2n-6	16.1±1.23^a^	12.6±1.07^b^	12.3±0.92^b^
20∶3n-6	0.9±0.07^b^	1.7±0.21^a^	1.9±0.20^a^
20∶4n-6	5.6±0.88^b^	7.1±0.69^a^	6.8±0.70^a^
22∶4n-6	1.5±0.09	1.4±0.14	1.5±0.21
Total	24.0±1.91	22.7±1.76	22.5±1.69
n-3 PUFA			
18∶3n-3	6.9±0.84^a^	4.0±0.37^b^	3.4±0.29^b^
20∶3n-3	3.0±0.23^a^	2.1±0.31^b^	1.8±0.13^b^
20∶4n-3	1.3±0.12^a^	0.9±0.13^b^	0.9±0.09^b^
20∶5n-3	0.6±0.08^c^	2.6±0.29^a^	1.8±0.15^b^
22∶5n-3	3.9±0.71^b^	7.2±0.99^a^	4.2±0.73^b^
22∶6n-3	4.7±0.48^b^	5.3±0.66^b^	12.5±0.85^a^
Total	20.4±1.30^b^	22.0±1.07^b^	24.6±1.51^a^

Values are means of three measurements. Values for each fatty acid with different superscripts differ significantly (*P*<0.05) between control (EGFP), sN3 and N3D4.

## Discussion

Here, we reported a new technology for enhanced production of DHA in mammalian cells. Mammals lack the Δ12 and Δ15 fatty acid desaturases responsible for converting oleic acid (18∶1*n*-9) into linoleic acid (18∶2*n*-6) and α-linolenic acid (18∶3*n*-3) and thus, are unable to biosynthesize polyunsaturated fatty acids (PUFA) *de novo* [27], [28]. Therefore, the ingested plant-derived 18∶2*n*-6 and 18∶3*n*-3 are essential dietary nutrients for mammals [27], [28]. Nevertheless, most of the ingested plant-derived 18∶2*n*-6 and 18∶3*n*-3 can not be converted to ARA, EPA or DHA but are lost to β-oxidation. Our results demonstrated that, by overexpression of Δ6 and Δ5 fatty acid desaturases, the ability of converting 18∶2*n*-6 to 20∶4n-6, 18∶3*n*-3 to EPA and DPA in mammalian cells was significantly elevated. This may be attributed to the fact that the overexpression of these desaturases disequilibrated the regulatory system of PUFA biosynthesis. We had studied that the overexpression of Δ6 and Δ5 fatty acid elongases (the most two important elongases involved in PUFA biosynthesis) in CHO cells, and found that they contributed little to elevating the level of any longer PUFA biosynthesized from 18∶2*n*-6 or 18∶3*n*-3 (unpublished data). We conclude that, among the fatty acid desaturases and elongases involved in PUFA biosynthesis in mammals, Δ6 and Δ5 desaturases are the key ones that contribute to production of longer PUFA.

The conversion of DPA to DHA is the rate-limiting step in the pathway of biosynthesis of DHA from ALA [29]. As shown in our results, even the overexpression of Δ6 and Δ5 fatty acid desaturases did not promote the conversion of DPA to DHA. They only led to an accumulation of high level of DPA in CHO cells. The results also confirmed that the Sprecher pathway in mammals had very low effciency to convert DPA to DHA. A few of lower organisms can directly convert DPA to DHA by utilizing their Δ4 desaturases activities. Some researchers believe that the rate of DHA synthesis could be faster in the more direct Δ4 pathway and that only requires endoplasmic reticulum than in the Sprecher pathway that involves peroxisomes, translocation of PUFA intermediates and limited fatty acid oxidation (a catabolic step). Δ4 desaturases have been demonstrated in some organisms including protozoan trypanosomes [30], the photosynthetic freshwater protist *Euglena gracilis* [31], marine microalgae *Pavlova lutheria* and *Thraustochytrids* [32], [33], and herbivorous, marine teleost fish, *Siganus canaliculatus* [34]. By heterologous expression in yeast, these Δ4 desaturases conferred on the yeast, the ability to convert DPA (22∶5n-3) to DHA. This paper has clearly demonstrated that, when heterologously expressed in CHO cells, the *S. canaliculatus* Δ4-desaturase can efficiently convert supplied DPA into DHA. More important, the proportions of DPA converted to DHA were about 36.3% in CHO cells expressing *S. canaliculatus* Δ4-desaturase, which is even much higher than that in transgenic yeast expressing this desaturase. This indicates the mammalian cells or even mammals are likely to accept these Δ4-desaturase activities from lower organisms. Significantly *S. canaliculatus* Δ4-desaturase activity could cooperate with Δ6 and Δ5-desaturase activities in co-transfected CHO cells with their gene constructs, directly and efficiently converting the accumulated DPA to DHA. Therefore, our strategy, by heterologous expression of *Siganus canaliculatus* Δ4 desaturase with overexpression of mice Δ6/Δ5-desaturase in mammalian cells, is very effective in high-level production of DHA from dietary ALA.

Production of DHA in land animals may be a good choice for mankind to meet the growing demand for this important LC-PUFA, and our results provide potential solution on this regard. Like humans, most land animals can accumulate DHA through their diet, but usually there is no adequate DHA in animal feeds. Adding DHA to animal feeds for the accumulation of it in animal bodies is expensive and unadvisable. Mammalian cells in culture, to a large extent, can simulate the process of PUFA biosynthesis in living animals. So our technology, by heterologous expression of *Siganus canaliculatus* Δ4 desaturases and overexpression of mice Δ6/Δ5-desaturases simultaneously, will probably produce high level of DHA in transgenic livestock carrying these gene constructs as in transfected CHO cells reported here. If such genetically modified land animals are successfully created and widely accepted by the public, DHA can be provided abundantly by these animals, which will probably change our views on the ordinary ‘unhealthy’ animal fat.

In conclusion, this work has demonstrated that mammalian cells can be genetically modified by transfection of an delta-4 desaturase that is heterologously expressed with concomitant overexpression of delta-5 and delta-6 desaturases to convert ALA to DHA efficiently, and provide a potential for creation of new land animal breeds who can produce abundant DHA in their related products.
